# Supplementary material for: Diversity of the Bacterial and Viral Communities in the Tropical Horse Tick, Dermacentor nitens, in Colombia
Source: Pathogens. 2023 Jul 16;12(7):942. doi: 10.3390/pathogens12070942 (PMC10384233; doi:10.3390/pathogens12070942)
Supplement: Supplementary file 1 [file pathogens-12-00942-s001.zip › pathogens-2442176-supplementary.pdf]

pathogens

MDPI

Article

Diversity of the Bacterial and Viral Communities in the Tropical Horse Tick, *Dermacentor nitens*, in Colombia

Andres F. Holguin-Rocha [1], Arley Calle-Tobon [2], Gissella M. Vásquez [3], Helvio Astete [3], Michael L. Fisher [3,4], Alberto Tobon-Castano [5], Gabriel Velez-Tobon [5], L. Paulina Maldonado-Ruiz [1], Kristopher Silver [1], Yoonseong Park [1,*] and Berlin Londono-Renteria [6,*]

[1] Department of Entomology, Kansas State University, Manhattan, KS 66506, USA; aholguin@ksu.edu (A.F.H.-R.); lpmaldonado@ksu.edu (L.P.M.-R.); ksilver@ksu.edu (K.S.)
[2] Grupo Entomologia Medica, Facultad de Medicina, Universidad de Antioquia, Medellin, Colombia; credoest@gmail.com
[3] U.S. Naval Medical Research Unit No. 6 (NAMRU-6), Bellavista, Callao, Peru; gissella.m.vasquez.ln@health.mil (G.M.V.); astetehelvio@gmail.com (H.A.); michael.l.fisher2@navy.mil (M.L.F.);
[4] Navy Warfare Development Center, Norfolk, VA, USA (Current Affiliation)
[5] Grupo Malaria, Facultad de Medicina, Universidad de Antioquia, Medellin, Colombia; alberto.tobon1@udea.edu.co (A.T.-C.); gabrielj.velez@udea.edu.co (G.V.-T.)
[6] School of Public Health and Tropical Medicine, Tulane University, New Orleans, LA, USA
* Correspondence: ypark@ksu.edu (Y.P.); blondono@tulane.edu (B.L.-R.)

Supplementary Material

Table S1. List of library sequences obtained in the metatranscriptomics study. Each pool comprises five *Dermacentor nitens* females. Refer to the Material and Methods section for more details about cleaning and mapping processes.

Library	Location	Raw Reads (Million)	Clean Reads (Million)	Mapped Reads against Horse and *Dermacentor* Ticks (Million)	Remaining Reads (Million)
Pool 01	Bolivar	15.2	14.2	1.6	12.55
Pool 02	Bolivar	14.1	13.2	5	8.18
Pool 03	Bolivar	15.9	14.8	5.2	9.67
Pool 04	Antioquia	19.2	17.8	2.5	15.37
Pool 05	Antioquia	18.1	16.8	6.1	10.58
Pool 06	Antioquia	15.7	14.7	5.1	9.54
Pool 07	Cordoba	16.6	15.6	1.9	13.7
Pool 08	Cordoba	15.3	14.4	5.9	8.4
Pool 09	Cordoba	22.1	21.3	17.2	4.19
Total		152.2	142.8	50.5	92.18

Table S2. Relative abundances of *Francisella*-Like Endosymbiont (FLE) captured in 16S sequencing. Heat map shows the percent abundance for each bacterial OTU identified as *Francisella/Francisellaceae*.

OTU Id	Bolivar	Antioquia	Cordoba
Otu001	61%	56%	59%
Otu010	18%	15%	22%
Otu002	10%	5%	3%

Table S3. The coverages of contigs for *Francisella*-Like Endosymbiont (FLE) genes. The coverages are shown by the $\log_{10}$ (transcript per million) in the metatranscriptome analysis. In the heat map, white is for low and red is for high frequency.

Sequence name	Bolivar	Antioquia	Cordoba

Contig_ORF_FLE_of_D.nitens_13	3.75	5.71	8.03
Contig_ORF_FLE_of_D.nitens_1	2.97	4.78	7.17
Contig_ORF_FLE_of_D.nitens_9	2.70	5.09	6.91
Contig_ORF_FLE_of_D.nitens_7	2.32	4.31	6.41
Contig_ORF_FLE_of_D.nitens_8	2.28	4.22	6.34
Contig_ORF_FLE_of_D.nitens_6	1.85	4.18	6.20
Contig_ORF_FLE_of_D.nitens_3	1.89	4.11	5.98
Contig_ORF_FLE_of_D.nitens_2	1.88	3.94	6.06
Contig_ORF_FLE_of_D.nitens_4	1.78	4.09	5.96
Contig_ORF_FLE_of_D.nitens_5	2.08	3.46	5.89
Contig_ORF_FLE_of_D.nitens_12	1.45	3.37	5.61
Contig_ORF_FLE_of_D.nitens_11	0.56	2.77	5.06
Contig_ORF_FLE_of_D.nitens_10	0.64	2.16	4.62

Table S4. The coverages of virus contigs shown by $\log_{10}$(transcript per million) for the metatranscriptome of *D. nitens*. In the heat map, white is for low and red is for high frequency.

Sequence name	Bolivar	Antioquia	Cordoba
D. nitens_Colombia_Flaviviridae_Polyprotein_6	5.96	7.28	7.50
D.nitens_Colombia_Chuviridae_RdRp_5	1.61	3.57	1.06
D. nitens_Colombia_Chuviridae_Glycoprotein_2	0.97	2.54	0.98
D. nitens_Colombia_Rhabdoviridae_RdRp_1	0.90	0.30	2.30
D. nitens_Colombia_Rhabdoviridae_RdRp_1	0.62	0.00	2.61
D. nitens_Colombia_Rhabdoviridae_RdRp_1	0.21	0.48	2.46
D. nitens_Colombia_Rhabdoviridae_RdRp_1	0.21	0.00	2.09
D. nitens_Colombia_Rhabdoviridae_Nucleocapsid_3	0.44	0.00	0.00
D. nitens_Colombia_Unclassified_Capsid_Protein_1	0.00	0.00	0.14

Table S5. Pairwise comparisons of the frequencies (transcript per million, TPM) in virus contigs among the ticks collected in different locations. The statistics were empirical Bayes estimation and exact tests (α = 0.05) in the pairwise comparisons of the frequencies of the paired two regions. $p <$ 0.05 is highlighted.

Sequence Name	Bolivar-Antioquia	Bolivar-Cordoba	Antioquia-Cordoba
	***P*-value**	***P*-value**	***P*-value**
Rhabdoviridae_Nucleocapsid_3	0.035130971	0.031573397	1
Rhabdoviridae_Polymerase_1C	0.123213517	0.221619581	0.028354078
Rhabdoviridae_Polymerase_1D	0.179418638	0.422800509	0.082456668
Rhabdoviridae_Polymerase_1B	0.30061269	0.789734348	0.219269539
Chuviridae_Polymerase_5	0.46796584	0.44991904	0.192016665
Chuviridae_Glycoprotein_2	0.484497213	0.794271421	0.366869359
Rhabdoviridae_Polymerase_1A	0.907602552	0.231983583	0.248901999
Flaviviridae_Polyprotein_6	0.983445117	0.982669254	0.999577095
Unclassified_Capsid_Protein_1	1	1	1

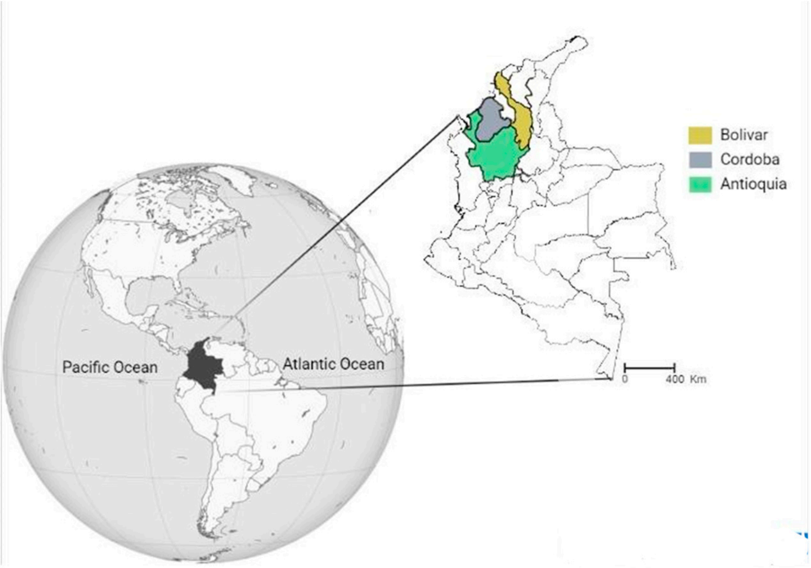

Figure S1. Locations of origin of horses from which ticks were sampled in this study. Colored zones represent the three different departments. Information was obtained from Instituto Geográfico Agustin Codazzi, and figure.

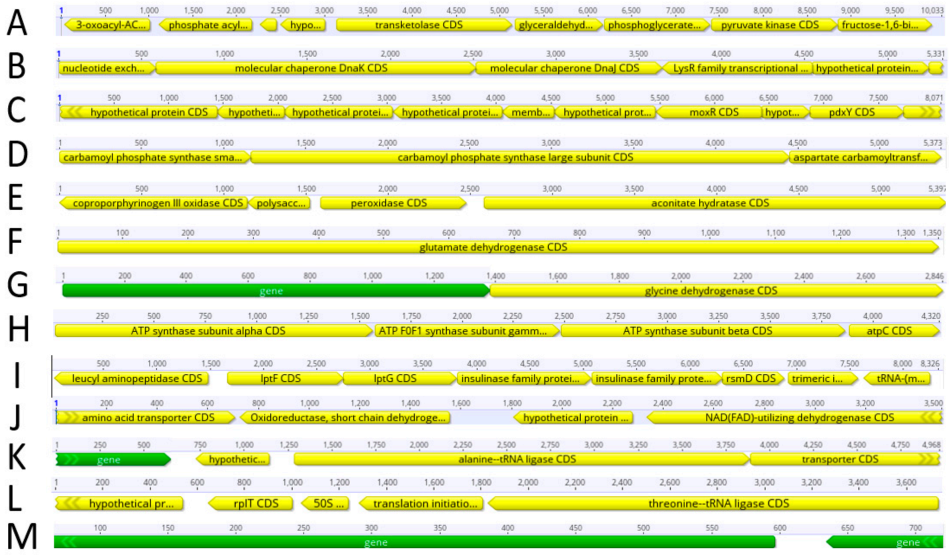

Figure S2. Graphical representation of the genes annotated for each contig identified as *Francisella*-Like Endosymbiont (FLE) obtained from the partially-fed *D. nitens* female pools. **(A)** represents the contig named Contig_ORF_D.nitens_FLE_1. **(B)** represents the contig named Contig_ORF_D.nitens_FLE_2. **(C)** represents the contig named Contig_ORF_D.nitens_FLE_3. **(D)** represents the contig named Contig_ORF_D.nitens_FLE_4. **(E)** represents the contig named Contig_ORF_D.nitens_FLE_5. **(F)** represents the contig named Contig_ORF_D.nitens_FLE_6. **(G)** represents the contig named Contig_ORF_D.nitens_FLE_7. **(H)** represents the contig named Contig_ORF_D.nitens_FLE_8. **(I)** represents the contig named Contig_ORF_D.nitens_FLE_9. **(J)** represents the contig named Contig_ORF_D.nitens_FLE_10. **(K)** represents the contig named Contig_ORF_D.nitens_FLE_11. **(L)** represents the contig named Contig_ORF_D.nitens_FLE_12. **(M)**represents the contig named Contig_ORF_D.nitens_FLE_13. The genes represented in yellow colorcorrespond to previously known annotated genes available on the NCBI website database, genes represented with green color are current non-annotated predicted genes obtained from thehomology-based search at the NCBI databases.

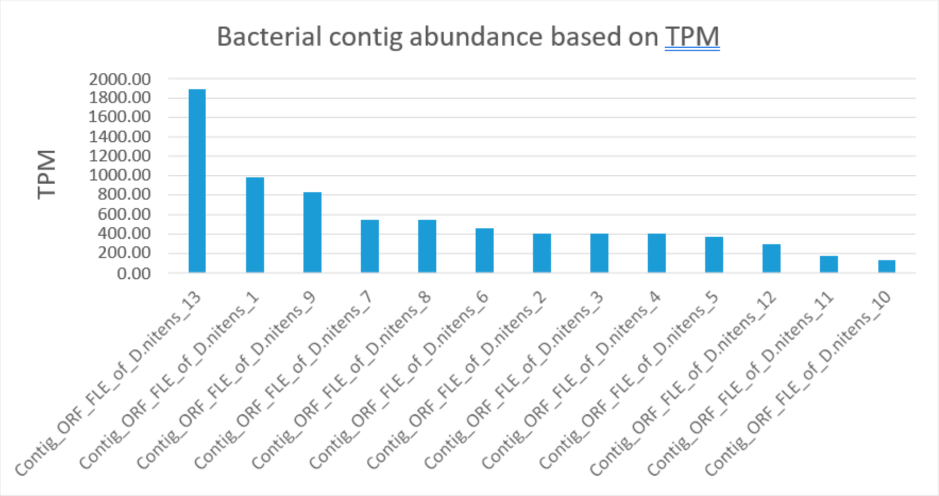

Figure S3. Abundance of FLE contigs in the metatranscriptome of *D. nitens*.

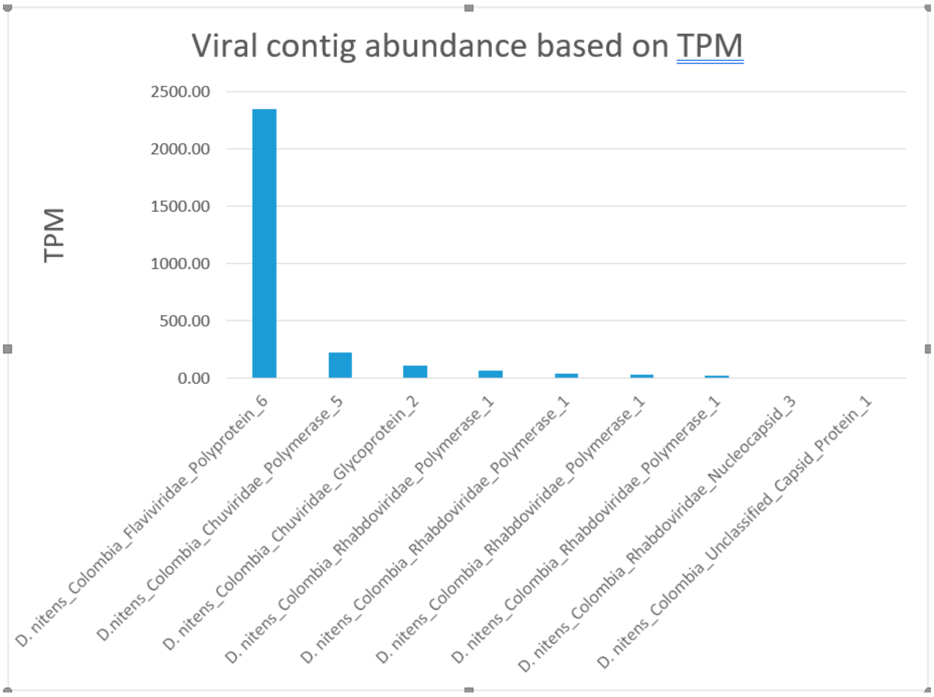

Figure S4. Abundance of virus contigs in the metatranscriptome of *D. nitens*.
